# Evaluation of a Weight Management Program for Veterans

**DOI:** 10.5888/pcd9.110267

**Published:** 2012-05-17

**Authors:** Alyson J. Littman, Edward J. Boyko, Mary B. McDonell, Stephan D. Fihn

**Affiliations:** Author Affiliations: Edward J. Boyko, Seattle Epidemiologic Research and Information Center, VA Puget Sound Health Care System, Seattle, Washington; Mary B. McDonell, Stephan D. Fihn, VHA Office of Informatics and Analytics, Office of Analytics and Business Intelligence, Seattle, Washington. Dr Boyko and Dr Fihn are also affiliated with the Department of Medicine, University of Washington, Seattle, Washington.

## Abstract

**Introduction:**

To improve the health of overweight and obese veterans, the Department of Veterans Affairs (VA) developed the MOVE! Weight Management Program for Veterans. The aim of this evaluation was to assess its reach and effectiveness.

**Methods:**

We extracted data on program involvement, demographics, medical conditions, and outcomes from VA administrative databases in 4 Western states. Eligibility criteria for MOVE! were being younger than 70 years and having a body mass index (BMI, in kg/m^2^) of at least 30.0, or 25.0 to 29.9 with an obesity-related condition. To evaluate reach, we estimated the percentage of eligible veterans who participated in the program and their representativeness. To evaluate effectiveness, we estimated changes in weight and BMI using multivariable linear regression.

**Results:**

Less than 5% of eligible veterans participated, of whom half had only a single encounter. Likelihood of participation was greater in women, those with a higher BMI, and those with more primary care visits, sleep apnea, or a mental health condition. Likelihood of participation was lower among those who were younger than 55 (vs 55-64), widowed, current smokers, and residing farther from the medical center (≥30 vs <30 miles). At 6- and 12-month follow-up, participants lost an average of 1.3 lb (95% confidence interval [CI], −2.6 to −0.02 lb) and 0.9 lb (95% CI, −2.0 to 0.1 lb) more than nonparticipants, after covariate adjustment. More intensive treatment (≥6 encounters) was associated with greater weight loss at 12 months (−3.7 lb; 95% CI, −5.1 to −2.3 lb).

**Conclusion:**

Few eligible patients participated in the program during the study period, and overall estimates of effectiveness were low.

## Introduction

An estimated 70% of veterans are overweight or obese, with a body mass index (BMI, in kg/m^2^) of 25.0 or more, consistent with the prevalence of overweight and obesity among demographically similar nonveterans (1-4). Weight loss as small as 5% can reduce the risk of chronic conditions associated with obesity (5). Participants in intensive lifestyle interventions such as those tested in the Diabetes Prevention Program and the Look Ahead trials achieved clinically significant weight loss (6,7). Mean weight losses in those trials were approximately 7% to 8% at 1 year, or 19 pounds (6,7). Translating these successful interventions into programs that can be disseminated widely and implemented in clinical and community settings is a key to reducing the prevalence of obesity.

The Department of Veterans Affairs (VA) administers the largest integrated health care system in the United States; it includes 152 medical centers and 804 community-based outpatient clinics (8). More than 8 million men and women were enrolled in the VA Health Care System in 2010, and approximately 6 million of them received health care in this system (8). To improve the care of veterans who are obese and overweight, VA created and disseminated a clinic-based weight management program, the MOVE! Weight Management Program for Veterans, beginning in 2005.

MOVE! is the largest clinically based weight management program in the United States. Little is known about the proportion of eligible VA patients (“candidates”) who participate in the program, the characteristics of participants, or the program’s effectiveness. The primary aims of this study were to 1) estimate participation in the program, including comparisons of veterans who did and did not participate, and 2) assess the program’s effectiveness in terms of weight change. Secondary aims were to evaluate effectiveness in subgroups and assess implementation and adoption of the program.

## Methods

We conducted an evaluation of the program in 1 of the 21 regional VA networks and used the RE-AIM framework (reach, effectiveness, adoption, implementation, and maintenance) for organizing our analysis, results, and interpretation, focusing mainly on reach and effectiveness (9). This framework emphasizes that for a program to be effective in the general population, evaluation of components other than efficacy is important.

### The MOVE! Weight Management Program for Veterans

The VA National Center for Health Promotion and Disease Prevention (NCP) developed MOVE! to provide a standardized format for weight management (10). NCP created the program and materials on the basis of published evidence and clinical practice guidelines from VA and non-VA committees and organizations, as well as other published studies (5,7,10-12).

To disseminate the program, NCP created handouts for patients, training modules for staff, curricula for group sessions, weight management assessment tools, and methods for electronic tracking of participation in program activities (10). Each facility was permitted to determine its own methods to identify patients for the program and the types and extent of offerings in the program.

The treatment components were intended to be individually tailored, integrated into each patient’s ongoing care, and implemented in clinics by multidisciplinary teams (eg, dietitians, physical and recreational therapists, social workers, and mental health professionals). Typically, during the first encounter, staff provide an overview of the program and instruct patients to complete a 23-item questionnaire on their diet, physical activity, health status, and prior weight loss attempts. An individualized report is then generated; it includes a list of recommended print-ready materials on nutrition, physical activity, and healthy behavior change available from the MOVE! website (www.move.va.gov/). MOVE! staff may also help patients set goals to change diet and physical activity at this initial encounter. Follow-up sessions may be group-based, one-on-one, or by telephone.

Pilot feasibility trials were conducted at 17 VA medical centers between October 2003 and December 2004. On the basis of lessons learned in the pilot testing, NCP revised the program components and materials and launched the program nationally in late 2005. VA leadership issued formal policy in early 2006 requiring weight management treatment to be available at all VA medical facilities (http://vaww.move.med.va.gov/).

### Data sources

Because there was no data source for national estimates of key variables, we performed these analyses using data from the VA Northwest Region database (Veterans Integrated Service Network [VISN 20]), which includes data on demographics vital signs, pharmacy use, and laboratory tests and clinical and administrative medical record data about use of outpatient and inpatient services. We obtained information on MOVE! participation and encounter type (ie, group, individual, or telephone) from the National Patient Care Database, which integrates enterprise-wide, patient-level administrative data related to the program. The institutional review boards of VA Puget Sound Health Care System and Portland VA Medical Center approved the study.

### Study population

We included patients who had a primary care encounter during the study period at any of the 8 VISN 20 facilities in Alaska, Idaho, Oregon, and Washington State (Figure). “Facility” refers to the main VA hospital and any affiliated satellite hospitals or community-based outpatient clinics (CBOCs); the number of patients at CBOCs was generally too small to analyze separately. For the cross-sectional (reach) analyses, the study period was between October 1, 2005, or the start of MOVE! implementation at each facility, whichever was later, and September 31, 2008. For the longitudinal (effectiveness) analyses, follow-up was until December 31, 2008, the most recent data available when this study was initiated. Because 1 facility did not launch a program until 2009, patients from this facility were excluded from all analyses. Two other facilities began program enrollment late in the study period and thus had few enrollees and limited follow-up time. Consequently, patients from these 2 facilities were considered for inclusion for the cross-sectional analyses only.

**Figure:**
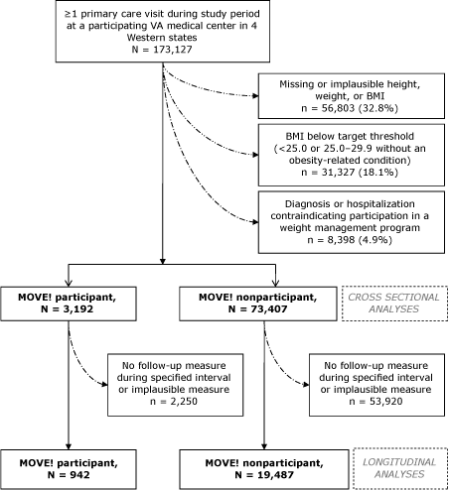
Flow diagram of VA Northwest patients included in cross-sectional and longitudinal analyses of the MOVE! Weight Management Program for Veterans. Visit had to be between October 1, 2005, or the patient’s facility’s initial MOVE! implementation date, whichever was later, and September 31, 2008. Participation was limited to patients aged 18 to 69. “Implausible” defined as <75 lb or >600 lb or an average weight loss or gain of >2 lb/wk *and* >50 lb overall, *or* >100 lb gain or loss, regardless of rate of change. “Contraindication” defined in Appendix. Numbers presented in the figure are for 12-month weight changes; numbers for 6-month weight changes were 951 participants and 17,139 nonparticipants. Abbreviation: BMI, body mass index.

To identify patients who were MOVE! candidates, we used BMI derived from heights and weights obtained during routine clinical encounters. We attempted to use heights measured during the same period as weights but used heights recorded as far back as January 1997. We used an iterative process to eliminate height, weight, and BMI measures that reflected probable data entry errors (Appendix).

Patients were considered candidates for MOVE! if they 1) had a BMI of at least 30.0, or 25.0 to 29.9 with an obesity-related condition and 2) were younger than 70 (because the program was not designed for older people). We excluded patients who had a medical condition that contraindicated weight management (Appendix). Patients were classified as participants if they had at least 1 encounter coded as related to MOVE!. Nonparticipants were defined as MOVE! candidates who had no MOVE!*-*related encounters.

### Assessment and statistical analyses of reach

We defined reach as the proportion of candidate veterans who participated in MOVE!. Representativeness was based on comparisons of participants to nonparticipants for key sociodemographic and health-related characteristics. To determine the independent associations between characteristics and participation, we created a multivariable logistic regression model.

### Assessment and statistical analyses of effectiveness

We attempted to obtain weights at the most relevant period while minimizing the number of patients dropped from analyses. For the longitudinal analyses, baseline weight for nonparticipants was the first recorded weight after program implementation at the patient’s facility (or a weight recorded up to 30 days prior) and for participants, a weight measured on the day of the patient’s first MOVE! encounter or up to 30 days prior.

For each person, we selected the weight closest to 183 and 365 days, respectively, after baseline for the 6-month and 12-month follow-up outcome measures. The range for the 6-month measures was 84 to 213 days (median: 168 and 169 days for nonparticipants and participants, respectively) and for the 12-month measures was 214 to 395 days (median: 349 and 350 days for nonparticipants and participants, respectively). We considered 12 weeks the minimum time necessary to result in meaningful changes in weight and thus the earliest follow-up time for the 6-month longitudinal analyses.

To compare changes in outcomes between participants and nonparticipants, we used multivariable linear regression. The follow-up values were included as the outcome and adjusted for the baseline measure and the duration of follow-up (in days), in addition to all factors significantly associated with participation. In our primary analyses, we evaluated participation as a dichotomous variable (yes/no). As a secondary analysis, we evaluated participation in terms of “intensity/dose” (ie, nonparticipation vs 1, 2 to 5, and ≥6 MOVE! encounters). Finally, to better understand factors associated with weight loss, we used multivariable logistic regression to determine associations between characteristics and clinically important weight loss in MOVE! participants, defined as at least 5% of baseline weight. Standard errors for multivariable analyses were adjusted for clustering of patients within facilities using a clustered sandwich estimator.

### Assessment of other measures

To assess adoption, we calculated the number of facilities that implemented MOVE! in the first year that it was disseminated. As a proxy measure of implementation, we assessed the average number of MOVE! encounters per person and the percentage of patients with only 1 vs 6 or more encounters, at the facility level, in the year following the initial visit.

## Results

### Reach

Of the 173,127 people who were potentially eligible for MOVE!, 76,599 were classified as MOVE! candidates and included in the cross-sectional analyses (Figure). The primary reasons for exclusion were missing or implausible weight, height, or BMI (n = 56,803); BMI below the threshold for inclusion (n = 31,327); and a diagnosis or hospitalization contraindicating participation in a weight management program (n = 8,398).

A total of 3,192 (4.2%) patients participated in MOVE!, and participation ranged by facility from 0.4% (Facility G) to 8.2% (Facility A) (Table 1). Participation was greater for sites that launched a MOVE! program in April 2006 or earlier.

**Table 1:** MOVE! Program Dates, Participation, and Encounters, by Facility, VA Northwest Region

Measures^a^	Facility						

	A	B	C	D	E	F	G
Month/year MOVE! program began	02/2006	04/2006	10/2005	05/2006^b^	10/2006	10/2007	05/2008
No. of candidate veterans	10,699	8,845	10,777	24,578	7,045	4,357	10,298
MOVE! participants, n (%)	872 (8.2)	587 (6.6)	727 (6.8)	777 (3.2)	170 (2.4)	23 (0.5)	36 (0.4)
No. of encounters, mean (IQR)	4.6 (2–6)	2.2 (1–2)	2.2 (1–3)	1.8 (1–2)	5.9 (2–7)	1.6 (1–2)	NC^c^
Participants with only 1 encounter, %	24.3	60.3	53.2	71.9	24.7	60.9	NC^c^
Participants with ≥6 encounters, %	25.6	9.0	5.5	5.4	31.8	0	NC^c^
Encounters that were group-based, %	20.5	93.1	3.6	72.7	95.7	0	90.1
Encounters that were telephone-based, %	50.7	1.5	1.5	9.0	3.9	0.5	9.4

Abbreviations: VA, Department of Veterans Affairs; IQR, interquartile range; NC, not calculated.

^a^ Estimates for enrollment and encounter data were calculated between October 1, 2005, or the date the facility began offering MOVE!, whichever was later, and September 31, 2008.

^b^ Estimates are based on data through May 2008 because no patients were coded with MOVE! identifiers between June 1, 2008, and December 31, 2008, because of an error.

^c^ Site began offering program <1 year before the end of follow-up; estimates could not be calculated.

After adjusting for all factors in Table 2, the following characteristics were associated with likelihood of participation at least 30% higher than the reference categories: female sex, BMI of 30.0 or more, 3 or more primary care visits, sleep apnea, and any mental health condition (including bipolar disorder, depression, or schizophrenia) (Table 2). Age younger than 55 (vs 55–64, the reference category), current smoking, being widowed (vs never married), and receiving care at a facility 30 miles or more from the patient’s home were associated with lower likelihood of participation.

**Table 2:** Characteristics of MOVE! Participants and Eligible Nonparticipants, VA Northwest Region

Characteristic	Nonparticipants (n = 73,407)^a^	Participants (n = 3,192)^a^	Multivariable-Adjusted^b^ Associations of Participation, OR (95% CI)
**Age, y**			
<40	9,595 (13.1)	327 (10.2)	0.6 (0.5–0.7)
40–54	24,239 (33.0)	1,049 (32.9)	0.7 (0.7–0.8)
55–64	30,117 (41.0)	1,399 (43.8)	1 [Reference]
65–69	9,456 (12.9)	417 (13.1)	1.0 (0.9–1.2)
Mean (SD)	53.6 (10.7)	54.7 (9.7)	NA
**Sex**			
Female	5,134 (7.0)	461 (14.4)	1.9 (1.7–2.2)
Male	68,273 (93.0)	2,731 (85.6)	1 [Reference]
**Race/ethnicity**			
White	43,076 (58.7)	1,826 (57.2)	1 [Reference]
African American	4,283 (5.8)	219 (6.8)	1.2 (1.1–1.5)
Native Hawaiian/Pacific Islander	1,034 (1.4)	40 (1.3)	0.9 (0.7–1.4)
American Indian/Alaska Native	739 (1.0)	24 (0.8)	0.8 (0.5–1.8)
Other/missing	24,275 (33.1)	1,083 (33.9)	1.0 (0.9–1.1)
**Facility**			
A	9,827 (13.4)	872 (27.3)	2.8 (2.5–3.2)
B	8,258 (11.3)	587 (18.4)	2.3 (2.1–2.7)
C	10,050 (13.7)	727 (22.3)	2.1 (1.8–2.4)
D	23,801 (32.4)	777 (24.3)	1 [Reference]
E	6,875 (9.4)	170 (5.3)	1.1 (0.9–1.3)
F	4,334 (5.9)	23 (0.7)	0.4 (0.3–0.6)
G	10,262 (14.0)	36 (1.1)	0.2 (0.1–0.3)
**% Service-connected^c^**			
Not service-connected	30,886 (42.1)	1,178 (36.9)	1 [Reference]
0–20	11,505 (15.7)	510 (16.0)	1.2 (1.1–1.3)
30–60	15,515 (21.1)	675 (21.2)	1.1 (0.99–1.2)
70–100	15,501 (21.1)	829 (26.0)	1.1 (1.0–1.2)
**Marital status**			
Never married	6,764 (9.2)	311 (9.7)	1 [Reference]
Married	42,179 (57.5)	1,803 (56.5)	0.9 (0.8–0.99)
Separated/divorced	22,110 (30.1)	987 (30.9)	0.9 (0.8–1.0)
Widowed	2,057 (2.8)	86 (2.7)	0.7 (0.5–0.9)
Unknown	297 (0.4)	5 (0.2)	NR^d^
**Served in support of wars in Iraq and/or Afghanistan**			
No	70,340 (95.8)	3,103 (97.2)	1 [Reference]
Yes	3,067 (4.2)	89 (2.8)	1.0 (0.8–1.3)
**Cigarette smoking status**			
Nonsmoker (never or former)	28,916 (39.4)	1,409 (44.1)	1 [Reference]
Current smoker	18,401 (25.1)	545 (17.1)	0.7 (0.6–0.8)
Unknown	26,090 (35.5)	1,238 (38.8)	0.9 (0.9–1.0)
**Body mass index, kg/m^2^**			
25.0–29.9	26,931 (36.7)	333 (10.4)	1 [Reference]
30.0–34.9	28,283 (38.5)	1,112 (34.8)	3.4 (3.0–3.9)
35.0–39.9	11,808 (16.1)	899 (28.2)	6.3 (5.5–7.2)
≥40.0	6,385 (8.7)	848 (26.6)	11.0 (9.6–12.7)
Mean (SD)	32.4 (5.3)	36.9 (6.6)	NA
**Facility type**			
Medical center	39,205 (53.4)	2,317 (72.6)	1 [Reference]
Community-based outpatient clinic (CBOC)	33,939 (46.2)	875 (27.4)	0.8 (0.7–0.8)
Unknown	263 (0.4)	0	NR^d^
**Distance to medical center or CBOC, miles**			
<30	37,000 (50.4)	2,100 (65.8)	1 [Reference]
≥30	36,300 (49.5)	1,089 (34.1)	0.6 (0.6–0.7)
Unknown	107 (0.1)	3 (0.1)	NR^d^
**Health care and chronic illnesses**			
**No. of primary care visits^e^**			
1 or 2	24,669 (33.6)	144 (4.5)	1 [Reference]
3 or 4	15,675 (21.4)	432 (13.5)	3.4 (2.8–4.2)
5–8	18,609 (25.4)	1,096 (34.3)	6.1 (5.1–7.4)
≥9	14,454 (19.7)	1,520 (47.6)	8.7 (7.2–10.5)
**Diabetes**			
No	47,520 (64.7)	1,682 (52.7)	1 [Reference]
Yes	25,887 (35.3)	1,510 (47.3)	0.9 (0.8–1.0)
**Coronary artery disease**			
No	61,933 (84.4)	2,567 (80.4)	1 [Reference]
Yes	11,474 (15.6)	625 (19.6)	1.0 (0.9–1.1)
**Hypertension**			
No	19,735 (26.9)	666 (20.9)	1 [Reference]
Yes	53,672 (73.1)	2,526 (79.1)	1.0 (0.9–1.1)
**Osteoarthritis**			
No	45,842 (62.5)	1,703 (53.4)	1 [Reference]
Yes	27,565 (37.6)	1,489 (46.7)	1.1 (0.99–1.2)
**Dyslipidemia**			
No	24,902 (33.9)	827 (25.9)	1 [Reference]
Yes	48,505 (66.1)	2,365 (74.1)	1.0 (0.95–1.1)
**Sleep apnea**			
No	61,487 (83.8)	2,181 (68.3)	1 [Reference]
Yes	11,920 (16.2)	1,011 (31.7)	1.3 (1.2–1.4)
**No. of comorbidities^f^**			
0	4,894 (6.7)	144 (4.5)	1 [Reference]
1	13,686 (18.6)	355 (11.1)	1.0 (0.8–1.2)
2 or 3	39,142 (53.3)	1,521 (47.7)	1.1 (0.9–1.3)
≥4	15,685 (21.4)	1,172 (36.7)	1.1 (0.9–1.4)
**Bipolar disorder**			
No	69,934 (95.3)	2,926 (91.7)	1 [Reference]
Yes	3,473 (4.7)	266 (8.3)	1.2 (1.1–1.5)
**Depression**			
No	45,780 (62.4)	1,637 (51.3)	1 [Reference]
Yes	27,627 (37.6)	1,555 (48.7)	1.2 (1.1–1.3)
**Schizophrenia**			
No	71,527 (97.4)	3,065 (96.0)	1 [Reference]
Yes	1,880 (2.6)	127 (4.0)	1.2 (0.96–1.5)
**Any mental health condition^g^**			
No	41,892 (57.1)	1,472 (46.1)	1 [Reference]
Yes	31,515 (42.9)	1,720 (53.9)	1.5 (1.4–1.6)

Abbreviations: VA, Department of Veterans Affairs; OR, odds ratio; CI, confidence interval; SD, standard deviation; NA, not applicable; NR, not reported.

^a^ Values are numbers (percentage) unless otherwise indicated; percentages may not sum to 100 because of rounding.

^b^ Adjusted for all other variables in the table. Standard errors used to calculate 95% CIs are based on a sandwich estimator that takes into account the clustering of individuals within facilities.

^c^ “Service connected” means that the disability was a result of disease or injury incurred or aggravated during active military service. Ratings are graduated according to the degree of the veteran’s disability on a scale of 0% to 100%, in increments of 10%. Zero percent is different than having no rating; it means that a disability exists and is related to the veteran’s service, but it is not so disabling that it entitles the veteran to compensation payments.

^d^ Estimates are not reported for categories with <10 participants.

^e^ Primary care visits from MOVE! implementation to end of follow-up.

^f^ Sum of comorbidities that indicate MOVE! eligibility, specifically, diabetes, coronary artery disease, hypertension, osteoarthritis, dyslipidemia, and sleep apnea.

^g^ Entered into separate logistic regression model in place of individual mental health conditions (bipolar disorder, depression, and schizophrenia).

### Effectiveness

Participants lost approximately 1 to 2 lb (0.2 to 0.3 kg/m^2^) during 6 to 12 months of follow-up (Table 3). After multivariable adjustment, mean weight losses in participants were significantly greater than in nonparticipants at 6 months (−1.3 lb) but not 12 months (−0.9 lb). Patients who had 6 or more encounters had significantly greater weight losses at 6-month and 12-month follow-up than nonparticipants (−2.6 lb; 95% CI, −3.8 to −1.5 and −3.7 lb; 95% CI, −5.1 to −2.3, respectively).

**Table 3:** Changes in Primary and Secondary Outcome Measures Among MOVE! Participants and Eligible Nonparticipants, VA Northwest Region

Measure	Mean (95% CI)		*P* Value

	Nonparticipants (n = 19,487)^a^	Participants (n = 942)^a^	
**Weight, lb**			
**Baseline**	223.3 (222.7 to 223.9)	252.3 (248.9 to 255.6)	<.001
**Follow-up**			
6 mo	224.4 (223.8 to 225.0)	250.5 (247.2 to 253.8)	<.001
12 mo	223.6 (223.0 to 224.2)	250.6 (247.2 to 253.8)	<.001
**Change**			
6 mo	0 (−0.2 to 0.1)	−2.1 (−2.8 to −1.5)	<.001
12 mo	0.3 (0.1 to 0.4)	−1.7 (−2.5 to −0.9)	<.001
**Adjusted^b^ Δ participants − Δ nonparticipants**			
6 mo	−1.3 (−2.6 to −0.02)		.048
12 mo	−0.9 (−2.0 to 0.1)		.07
**Body mass index, kg/m^2^**			
**Baseline**	32.2 (32.1 to 32.3)	36.8 (36.4 to 37.2)	<.001
**Follow-up**			
6 mo	32.4 (32.3 to 32.4)	36.5 (36.1 to 36.9)	<.001
12 mo	32.2 (32.2 to 32.3)	36.6 (36.1 to 37.0)	<.001
**Change**			
6 mo	0 (−0.02 to 0.02)	−0.3 (−0.4 to −0.2)	<.001
12 mo	0 (0.02 to 0.06)	−0.2 (−0.4 to −0.1)	<.001
**Adjusted^b^ Δ participants − Δ nonparticipants**			
6 mo	−0.2 (−0.4 to −0.01)		.04
12 mo	−0.1 (−0.3 to 0.001)		.05

Abbreviations: VA, Department of Veterans Affairs; CI, confidence interval; BMI, body mass index.

^a^ Data were available to assess 6-month change measures for 17,139 nonparticipants and 951 participants. Mean baseline weight and BMI in nonparticipants was 224.4 lb (95% CI, 223.8–225.0 lb) and 32.4 kg/m^2^ (95% CI, 32.3–32.4 kg/m^2^), respectively. Mean baseline weight and BMI in participants was 252.6 lb (95% CI, 249.3–255.9 lb) and 36.8 kg/m^2^ (95% CI, 36.4–37.2 kg/m^2^), respectively.

^b^ Multivariable adjusted analyses included the following covariates: baseline value for measure, days between baseline and follow-up measurements, age at baseline (<40, 40–54, 55–64, 65–69), sex, race (white, black, Native Hawaiian/Pacific Islander, American Indian/Alaska Native, other), marital status (never married, married, divorced/separated, widowed), facility (5 sites), service connectedness (not service-connected, 0%–20%, 30%–60%, 70%–100%), cigarette smoking status (never or former, current, unknown), BMI at baseline (continuous), medical center or community-based outpatient clinic (CBOC), distance to medical center or CBOC (<30 miles, ≥30 miles), number of primary care visits during follow-up (1 or 2, 3 or 4, 5–8, ≥9), hypertension, osteoarthritis, dyslipidemia, sleep apnea, and mental illness. Standard errors were adjusted for clustering of participants in facilities using a clustered sandwich estimator.

There were no consistent associations between facility and clinically important weight loss; for example, although the likelihood of clinically important weight loss at 6 months was approximately 4 times greater at Facility B than Facility D, no such association was apparent for 12-month weight loss. Women (vs men) and those with 2 or more comorbidities (vs none) had a lower likelihood of clinically important weight loss, while greater BMI was associated with higher likelihood of clinically important weight loss.

### Implementation

The mean number of encounters during follow-up was 3.0 and varied among facilities (range, 1.6–4.6) (Table 1); 49.6% of participants had only a single encounter (range among facilities, 24.3%–71.9%), while 13.1% had 6 or more encounters (range among facilities, 0%–31.8%). Additionally, the percentage of encounters that were group-based differed among facilities (0%–95.8%).

### Adoption

Five of the 8 VISN 20 facilities launched a MOVE! program by October 2006 (within 12 months of national implementation), whereas 3 facilities did not begin offering MOVE! until at least 2 years after the program was implemented nationally.

## Discussion

This study demonstrated that only a small proportion (<5%) of veterans who were candidates for MOVE! participated. Participation was associated with reductions in weight, although the reductions were small and of questionable clinical significance, albeit comparable in magnitude to several other “real world” implementation studies conducted in similar settings (13,14).

Women were more likely to participate than men but were less likely to have clinically important weight loss. BMI was strongly and positively associated with both participation and clinically important weight loss. Although participants had more primary care visits and obesity-related conditions, including sleep apnea, than nonparticipants, participants with more comorbidities were less likely to lose at least 5% of their weight than those with fewer comorbidities or primary care visits. One novel finding was a greater likelihood of participation among veterans with a mental health condition, although there was no evidence of greater effectiveness. People with a mental health condition in the current study also had more obesity-related conditions (eg, heart disease, diabetes, sleep apnea). They may have been viewed by their health care provider as at greater risk for the consequences of obesity, and because of their comorbidities and likely greater contact with health professionals, may have had more opportunities to be offered enrollment in this program. These findings suggest that high-risk participants, such as those with mental health conditions, are interested in weight management. Determining methods to engage them and help them achieve weight management goals is an area for future research.

Evaluating the effectiveness of MOVE! is challenging because it is not clear that the program was implemented as intended. Sustained and intensive treatments are associated with better outcomes (12). Participants in the Diabetes Prevention Program and Look Ahead trials met with interventionists an average of 23.6 and 35.4 times, respectively, in the first year (6,15), nearly an order of magnitude more than was observed for VA Northwest Region patients.

We observed large variability in implementation across facilities. This variability is in part a product of the VA system because decisions on resource allocation are made at the local level. Some facilities in the study region offered only a single educational seminar each month, while other facilities offered 12-session group-based classes 2 or more times per week in addition to ongoing weekly maintenance sessions. Because of the small number of facilities in our study sample and the heterogeneity among them, it was not possible to evaluate associations between facility-level factors and participation or outcomes; other groups are in the process of doing so (16). This area of research may lead to improved reach and effectiveness.

Our study had several limitations. Patients were not randomized into MOVE!. Thus, confounding factors related to motivation to lose weight may explain some or all of the weight loss differences observed. Second, we were unable to assess clinical eligibility for MOVE! or weight change in a large number of patients because of missing data; as a result, participation rates may have been overestimated because many of those without weights and/or heights recorded in the medical record were likely candidates for the program. Conversely, use of administrative databases permitted us to include a large sample of both program participants and nonparticipants and to assess changes in measured rather that self-reported weight. Third, the findings from the Northwest region during the study period may not generalize to other areas of the country because of differences in patients, local implementation, and resources allocated to the program. Fourth, information was not available on weight loss activities unrelated to MOVE!, which may have differed between participants and nonparticipants. Finally, we were limited in our ability to assess other aspects of the RE-AIM framework, including direct measures of implementation, organizational factors, and maintenance (9).

This study focused on evaluation of MOVE! in the first few years after implementation. In 2008, the VA introduced a national overweight/obesity screening performance measure that requires providers to screen patients annually for obesity using BMI, provide obesity risk counseling, and offer comprehensive weight management treatment when appropriate (11). This measure may force facilities to reevaluate and reconfigure their existing MOVE! programs to better serve the increased number of veterans who will be offered treatment.

MOVE! participation in the 4 Western states studied in this evaluation was associated with small weight losses; weight losses were greater and suggested clinically important benefits in patients who received more intensive treatment (ie, ≥6 encounters). Although the findings for more intensive treatment are encouraging, only a small fraction of participants achieved this level of intensity, resulting in a small overall impact of the program. The lack of resources available to implement the program was likely a major contributing factor to the low participation and the limited evidence of effectiveness (17). Ideally, future evaluations will collect information from a national sample of community-based outpatient clinics and medical centers to determine both facility-level and individual-level factors associated with better outcomes. One benefit of the implementation of widespread screening for obesity in the VA is that it will be possible to assess MOVE! candidacy and effectiveness in a greater proportion of VA patients. Such evaluations will provide valuable information about how to increase the efficacy of the program to improve the health and well-being of overweight and obese veterans.

## Appendix

### Algorithms to identify measures that were implausible and/or erroneous vital sign values

For weight, height, and body mass index, we first removed biologically implausible values (weight <75 lb or >600 lb, height <49 in or >94 in, and body mass index >80 kg/m^2^) (1,2). Next, we applied algorithms to identify measures that were plausible but appeared to be erroneous on the basis of a review of all recorded weights and heights during the relevant time period. After reviewing records that had large standard deviations (SDs) (explained in more detail below), we used the algorithm that follows to exclude values that were likely erroneous while keeping values that were plausible. We excluded any weight measurements that met the following 2 criteria: 1) the difference between the mean weight and weight in question was greater than the SD and 2) the SD was greater than 10% of the mean. For example, 1 participant’s weight in pounds was recorded as 300 and 160 lb, both measured on December 7, 2005, 310 lb measured on June 12, 2006, 276 lb measured on August 8, 2006, 291 lb measured on August 15, 2006, and 291 lb measured on September 13, 2007, resulting in mean (SD) of 271.3 (55.7) lb. The weight of 160 lb recorded on December 7, 2005, was considered erroneous and dropped because the difference between the index weight and mean weight was greater than the SD (271 − 160 = 113.3 lb) and the SD was greater than 10% of the mean of all weights ([55.7/271.3] × 100 = 20.5%). Using a similar method, we considered a participant’s height measurement to be erroneous if 1) the difference between the mean height and height in question was greater than the SD and 2) the SD was greater than 2.5% of the average height. The most deviant height was dropped, and the same algorithm was rerun to identify any additional implausible height values. Participants with at least half of remaining heights flagged as implausible were dropped from analyses. Only 0.2% of measured weights and 0.99% of measured heights were considered erroneous and excluded after applying these rules.

For the secondary outcomes, the following values were considered to be implausible: high-density lipoprotein (HDL) cholesterol <10 or >120 mg/dL, LDL cholesterol <30 or >300 mg/dL, systolic blood pressure <60 or >250 mm Hg, and diastolic blood pressure <40 or >140 mm Hg.

### Conditions resulting in exclusion from analyses

Participants with *International Classification of Diseases, Clinical Modification, Ninth Revision* (ICD-9-CM) diagnosis codes indicating the presence of 1 or more of the following conditions were not considered eligible for a weight management program: HIV; progressive central nervous system infections; organic brain syndromes or dementias; anorexia; anterior horn cell disease (including amyotrophic lateral sclerosis); Huntington’s disease; cirrhosis; dialysis; congestive heart failure; chronic obstructive pulmonary disorder; neurological disorders; septicemia; peritonitis; hepatitis with hepatic coma; transplant surgery; or residing in a nursing home, hospice, residential, or adult day health care. In addition, participants with a hospitalization within 30 days before or after their last recorded primary care visit were ineligible. We excluded participants who were pregnant in the prior year, had a cancer diagnosis combined with oncology codes indicating active treatment, or had 3 or more admissions for day hospital or intensive care management. Oncology codes indicating active treatment were 141.0–208.9x (except 140–140.9 and 173.0–173.9) plus 2 or more oncology Stop Codes (XRT 149, Onc 316, ChemoRX 330, 431).
